# Genetic deletion of the Histone Deacetylase 6 exacerbates selected behavioral deficits in the R6/1 mouse model for Huntington’s disease

**DOI:** 10.1002/brb3.361

**Published:** 2015-06-24

**Authors:** Alienor Ragot, Susanna Pietropaolo, Jean Vincent, Pauline Delage, Hongyu Zhang, Bernadette Allinquant, Xavier Leinekugel, André Fischer, Yoon H Cho

**Affiliations:** 1 Institut de Neurosciences Cognitives et Intégratives d’Aquitaine, CNRS UMR 5287Avenue des Facultés, 33405, Talence Cedex, France; 2 University of Bordeaux146, rue Léo-Saignat, 33077, Bordeaux, France; 3 Interdisciplinary Institute for Neuroscience, CNRS UMR 529733000, Bordeaux, France; 4 Faculté de Médecine, Laboratoire INSERM, UMR 894- Université Paris Descartes, Sorbonne Paris CitéParis, France; 5 Neurocentre Magendie146, rue Léo-Saignat, 33077, Bordeaux, France; 6 Department for Psychiatry and Psychotherapy, University Medical Center GöttingenGrisebachstr. 5, 37077, Göttingen, Germany; 7 German Center for Neurodegenerative Diseases (DZNE) GöttingenGrisebachstr. 5, 37077, Göttingen, Germany

**Keywords:** Brain-derived neurotrophic factor, cognitive behavior, epigenetics

## Abstract

**Introduction:**

The inhibition of the Histone Deacetylase 6 (HDAC6) increases tubulin acetylation, thus stimulating intracellular vesicle trafficking and brain-derived neurotrophic factor (BDNF) release, that is, cellular processes markedly reduced in Huntington’s disease (HD).

**Methods:**

We therefore tested that reducing HDAC6 levels by genetic manipulation would attenuate early cognitive and behavioral deficits in R6/1 mice, a mouse model which develops progressive HD-related phenotypes.

**Results:**

In contrast to our initial hypothesis, the genetic deletion of HDAC6 did not reduce the weight loss or the deficits in cognitive abilities and nest-building behavior shown by R6/1 mice, and even worsened their social impairments, hypolocomotion in the Y-maze, and reduced ultrasonic vocalizations.

**Conclusions:**

These results weaken the validity of HDAC6 reduction as a possible therapeutic strategy for HD. The data are discussed in terms of additional cellular consequences and anatomical specificity of HDAC6 that could explain these unexpected effects.

## Introduction

Huntington’s disease (HD) is a fatal and hereditary neurodegenerative disease caused by the mutation of the *Huntingtin* gene. The mutation results in an abnormal expansion of polyglutamine tract in the huntingtin protein (The Huntington’s Disease Collaborative Research Group, 1993) and HD is observed in individuals with more than 36–39 CAG repeats. Chorea and motor impairments are accompanied by weight loss, deficits in cognitive functions (e.g., cognitive flexibility, visuospatial working memory), and multiple psychiatric symptoms (e.g., anxiety, depression, deficits in social behaviors) (Folstein and Folstein 1983; Swerdlow et al. 1995; Lawrence et al. 1998; Barquero-Jimenez and Gomez-Tortosa 2001; Craufurd et al. 2001; Paulsen et al. 2001; Snowden et al. 2002; Duff et al. 2007; Paradiso et al. 2008; Tabrizi et al. 2009). All symptoms appear usually in midlife and their severity increases progressively, ultimately leading to death (Conneally 1984). While the disease-causing mutation is expressed throughout neuronal and nonneuronal cells, striatal and cortical neurons undergo the earliest and most severe degeneration.

No treatment is yet available. However, among several approaches, histone deacetylases (HDAC) have emerged as potential therapeutic targets to treat neurodegenerative diseases including HD (Graff et al. 2012). More precisely, it has been shown that HDAC inhibitors (i.e., TSA and SAHA) exert neuroprotective effects in cell cultures by correcting mutant huntingtin-induced deficits in vesicular transport of brain-derived neurotrophic factor (BDNF) (Gauthier et al. 2004; Dompierre et al. 2007). This occurs via enhancing acetylation of alpha-tubulin, which plays an important role in BDNF transport. Because alpha-tubulin is one of major substrates of HDAC6 (Hubbert et al. 2002), neuroprotective effects have been tested *in vivo* by the genetic deletion of HDAC6 in R6/2 mice, which expresses 150 repeats (Bobrowska et al. 2011). This study failed to demonstrate the beneficial effects of HDAC6 gene deletion on behavioral HD phenotypes as well as BDNF transport, although tubulin acetylation was significantly increased. However, the effects of HDAC6 deletion were assessed only on motor functions and R6/2 mice express a high number of CAG repeats (more than 150 repeats) (Mangiarini et al. 1996) resulting in a juvenile onset and rapid progression of the disease. Hence, it is possible that these conditions were not optimal for observing the protective effects of the HDAC6 inhibition, especially on those behavioral (cognitive and psychiatric-like) perturbations, which precede the motor deficits in HD transgenic mice by several weeks or months (Lione et al. 1999; Grote et al. 2005; Nithianantharajah et al. 2008).

We therefore performed a study to specifically test whether the reduction in HDAC6 levels may rescue early cognitive and psychiatric symptoms in motor presymptomatic R6/1 mice. R6/1 mice display milder and slower phenotypes relative to R6/2 mice: first nuclear aggregates appear at 8–9 weeks, cognitive changes at 8–12 weeks, deficits in social behaviors, and motor coordination at 12–20 weeks of age, while body weight loss appears at 16–22 weeks (Naver et al. 2003; Nithianantharajah et al. 2008). Here, we assessed the effects of genetic deletion of HDAC6 on the progression of these early behavioral HD-like phenotypes by testing double mutant for HDAC6 and HD, and their single mutant and wt controls at 2 and 3 months of age. In addition to the known phenotypes, we evaluated a fine sensorimotor innate behavior, that is, nest-building behavior, which has been shown to be impaired in mouse models of neurodegenerative diseases (Cramer et al. 2012; Paumier et al. 2013).

## Materials and Methods

### Subjects

Subjects were male F1 mice derived from crossbreeding between R6/1 transgenic mice (Mangiarini et al. 1996) (C57bl/6 background, strain number: 006471; Jackson Laboratory, Bar Harbor, ME) and HDAC6 Knockout mice (sv129 background) (Govindarajan et al. 2013). Crossbreeding was performed using heterozygous HDAC6 females and hemizygous R6/1 males. At weaning pups were group-housed with their same-sex littermates (3–5/cage). Tail samples were used for PCR for both R6/1 and HDAC6 genotyping. While R6/1 mice were hemizygous for mutant HD gene, nullzygotes were used for HDAC6 mutation because HDAC6 gene is located on X-chromosome (Means et al. 2000). Male progeny from the crossbreeding was either wild type for both genes (HDAC6-WT+HD-ntg: *n* = 8), or HDAC6 wild-type and R6/1 transgenic (HDAC6-WT+R6/1: *n* = 8), or HDAC6-KO and nontransgenic for HD gene (HDAC6-KO+HD-ntg: *n* = 8), or HDAC6-KO and R6/1 transgenic (HDAC6-KO+R6/1, double mutant mice, *n* = 12). All mice were 2 month-old at the beginning of behavioral experiments.

NMRI mice (13 adult virgin females of 3–4 months of age) were used as stimulus animals in the social interaction test. They were purchased from Janvier (Le Genest-Saint-Isle, France), housed in groups of 4–5 individuals, and left undisturbed for 2 weeks before being used for testing. Each stimulus animal was employed multiple times in the same experiment (2–3 times in total), but different mice were used at each testing point. The use and order of presentation of the stimulus animal were balanced across genotypes. The NMRI strain is commonly employed in studies of social behavior, because of its high levels of sociability (D’Amato and Pavone 1996).

The experimental and stimulus animals were maintained in separate male and female colony rooms under temperature (22°C) and humidity-controlled (55%) conditions with a 12:12 h light–dark cycle (light on at 7 am). All testing occurred during the light phase unless otherwise mentioned. All experimental procedures were approved by the Institutional Animal Care and Use Committee: Comité d’Ethique pour l’Expérimentation Animale Bordeaux, and were in accordance with the European Communities Council Directive of 24 November 1986 (86/609/EEC).

### Behavioral procedures

The four groups of mice (HDAC6-WT+HD-ntg, HDAC6-WT+R6/1, HDAC6-KO+HD-ntg, and HDAC6-KO+R6/1) were submitted to a sequence of behavioral tests at 2 and 3 months of age. At each age, mice were first tested on spatial recognition in the Y-maze (day 1), followed by direct social interaction with a female (day 2). At the end of the second day, mice were given nesting materials and nest-building behavior was assessed on day 3. For these tests, mice were housed in standard individual holding cages for either 1 h (Y-maze and social interaction) or 17–18 h prior to testing (nest building).

### Body weight and tail clasping

Mice were weighted at 6, 8, 10, 12, and 14 weeks of age. The tail test was used to detect the abnormal clasping of the hind limbs (Mangiarini et al. 1996), and was used to ensure motor presymptomatic status of R6/1 mice. Mice were suspended by the tail for 10 s; if the mouse acquired a locked body position the result was scored as positive.

### Spatial recognition in Y-maze

A gray plastic Y-maze was used for spatial recognition memory test. The maze was placed on a table 80 cm high and located in a room containing extramaze visual cues. The three arms (42 × 8 × 15 cm) of the Y-maze were similar in appearance and dimension, and spaced at 120° from each other. The mouse’s locomotion was tracked and analyzed via a camera placed above the maze using Ethovision 9 (Noldus Technology, Wageningen, The Netherlands).

Mice were assigned two arms (start and familiar arms) to which they were exposed during the first phase of the test (sample phase). The remaining third arm, blocked by a transparent door placed at the entrance, constituted the novel arm during the second phase (test phase). Allocation of arms (start, familiar, and novel) was counterbalanced within each experimental group and it was varied between the two testing points. During the sample phase, mice were placed at the end of the designated start arm and allowed to explore freely both the start and the other unblocked (familiar) arm for 5 min. Mice were then removed from the maze and returned to the waiting cage for 10 min of retention interval, before the test phase began. During the test phase, the door blocking the “novel arm” was removed. Mice were placed at the end of the same start arm and allowed to explore the entire maze for 2 min. Timing of the 2 min test phase period was initiated only once the mouse had left the start arm. In the interval between the sample and the test phase, the apparatus was cleansed with alcohol and water to remove odor residue.

Time spent in each arm of the maze was analyzed during both phases of the experiment. Performance on the test phase was evaluated by time spent in the novel arm in comparison with the other arms. A preference index was calculated as the time spent in the novel arm divided by the time spent in all three arms × 100.

### Social interaction and ultrasonic vocalizations

Direct social interaction was assessed in standard rodent cages in which the experimental animals were housed for 1 h for habituation. An unfamiliar stimulus mouse (3–4 month-old female NMRI) was then introduced into the testing cage and left there for 3 min. During the test, an ultrasonic microphone (UltraSoundGate CM16, Avisoft Bioacoustics, Berlin, Germany) sensitive to frequencies of 15–180 kHz with a flat frequency response (±6 dB) between 25 and 140 kHz, was suspended 10 cm above the cage lid. It was connected via an external audio interface (USG 116, Avisoft Bioacoustics, Berlin, Germany) to a personal computer, where acoustic data were recorded at 250 kHz in 16-bit format and stored as wav files for subsequent analysis. Vocalizations were then analyzed with Avisoft SASLab Pro (Version 5.013, Avisoft, Berlin, Germany) as previously described (Pietropaolo et al. 2011).

Testing sessions were recorded and videos were analyzed using Observer XT (version 7, Noldus, The Netherlands), considering only the experimental animal. An experimenter who was unaware of the genotype of the animals scored the time spent performing social affiliative behaviors and nonsocial activities. Affiliative behaviors were defined as sniffing the head, snout, or anogenital region of the partner, contact of the body, allogrooming (grooming the partner), traversing the partner’s body by crawling over/under (Terranova et al. 1993; McFarlane et al. 2008; Pietropaolo et al. 2011). Nonsocial activities included rearing (standing on the hind limbs sometimes with the forelimbs against the walls of the cage), selfgrooming, and digging.

### Nest-building behavior

Nesting behavior was observed by providing a sheet of the laboratory cleansing paper in individually housed mice approximately 1–2 h prior to the onset of the dark phase. The nest quality was assessed the next morning (10.00–11:00) on a rating scale of 1–5 (1: paper not touched, 5: perfect and tridimensional nest, cf. Fig. 4A and B) according to the standard scoring technique (Deacon 2006).

### Tubulin acetylation and BDNF

The protein levels of acetylated tubulin and BDNF in brain tissue were analyzed by western blot. For that, a subset of mice (3 HDAC6-WT+HD-ntg, 5 HDAC6- WT+R6/1, 5 HDAC6-KO+HD-ntg, and 6 HDAC6-KO+R6/1) used in the behavioral study was euthanized at 14 weeks of age, their cortex and striatum were dissected and kept at −80°C. Samples were homogenized in 20 *μ*L of lysis buffer (20 mmol/L Tris Ph 7.6, 137 mmol/L NaCl, NP40 1%, glycerol 10%, protease inhibitor cocktail tablets (Roche, Meylan, France, 05892 970 001)) per mg of tissue. After homogenization, samples were centrifugated at 15,000 rpm for 20 min. Supernatant proteins were loaded in precast gel 4-15% Gradient (BioRad, Marnes-la-Coquette, france) and transferred to Immunobilon-P membranes (Millipore, Fontenay sous Bois, France). Blots were blocked in 5% nonfat dry milk in TBS-T (25 mmol/L Tris pH 7.6, 137 mmol/L NaCl, 27 mmol/L KCl, 0.01% Tween) and then incubated with primary antibodies 1:2000 for acetylated alpha-tubulin (Santa Cruz Biotechnology, Nanterre, France, Sc-23950) and 1:500 for BDNF (Santa cruz biotechnology, Nanterre, France, Sc-546). Blots were incubated with 1: 10,000 of anti-rabbit IgG Horse Radish Peroxydase (HRP)-conjugated (Jackson, Montlucon, France, 211-032-171) and 1:10,000 of anti-mouse IgG HRP-conjugated (Jackson, Montlucon, France, 115-035-174), for BDNF and tubulin, respectively, and developed by SuperSignal West Femto (Thermo Scientific, Villebon sur Yvette). As loading controls, a monoclonal antitubulin antibody (1:5000; Sigma, Saint-Quentin Fallavier, France, T9026) was used.

### Statistical analyses

Behavioral data, otherwise stated, were analyzed using three-way ANOVA with HD and HDAC6 genotypes as between-group factors, and age as within-group factor followed by post hoc comparisons using Fisher’s test. Due to technical problems, the data from one HDAC6-KO+R6/1 mouse, one HDAC6-WT+HD-ntg, and one HDAC6-KO+HD-ntg could not be analyzed for spatial recognition and 2 HDAC6-KO+HD-ntg at 2 months for social behavior. The same three-way ANOVA was used for separate habituation and test phases for spatial recognition. Data for the number of USVs were submitted to square-root transformation to better conform to the assumption of parametric analysis. Data were presented as mean ± SEM. The nest building as well as biochemical data were analyzed using nonparametric Mann–Whitney *U* test because of the nonnormal distribution of data and the small number of samples, respectively.

## Results

### Weight and clasping

**Figure 1:**
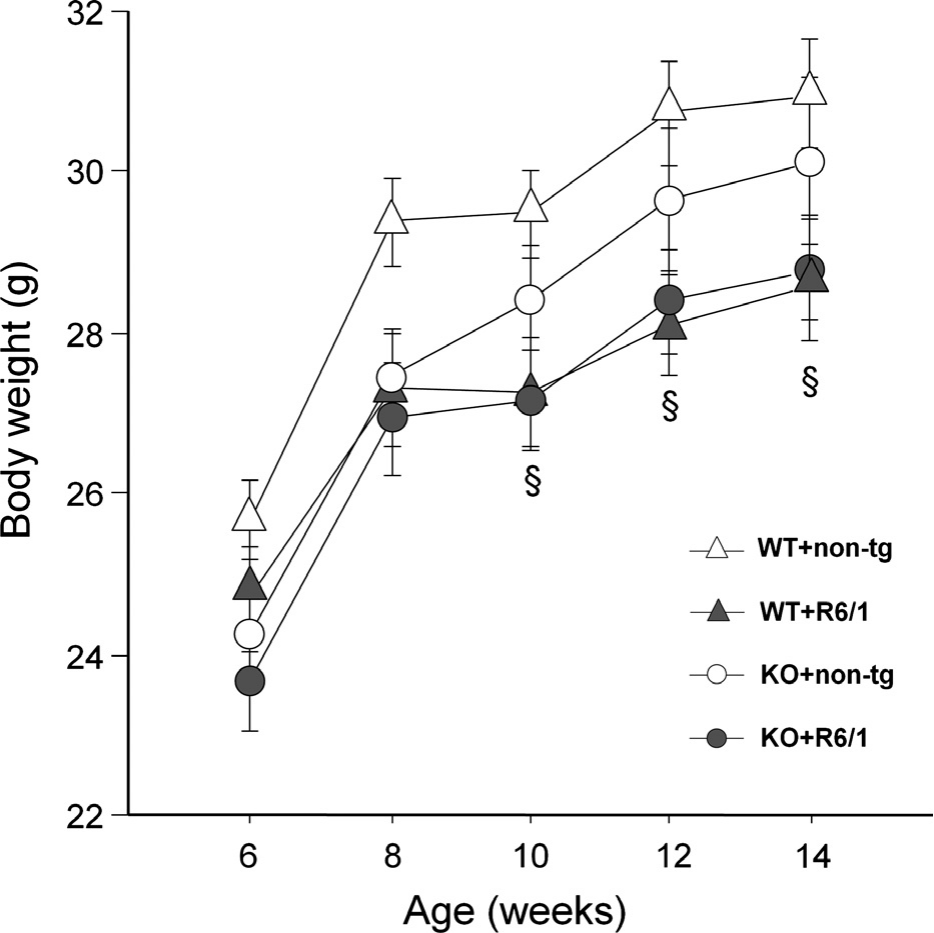
Effects of HDAC6 knockout on body weight in R6/1 mice. ^§^R6/1 main genotype effect: *P* < 0.05.

As expected, body weight increased with age (main age effect: *F*(4, 128) = 176.06, *P* < 0.0001; Fig.1), but this effect was reduced in R6/1 mice, irrespective of their HDAC6 genotype (age × R6/1 genotype interaction: *F*(4, 128) = 2.86, *P* < 0.05). R6/1 animals were significantly smaller than their wt littermates (main R6/1 genotype effect: *F*(1, 32) = 5.93, *P* < 0.05), and their body weight loss started at 10 weeks of age (post hoc: *P* < 0.05). There was no effect of HDAC6 genotype (main HDAC6 genotype effect: *F*(1, 32) = 1.26, n.s.), nor R6/1 × HDAC6 genotype interaction (*F*(1, 32) < 1, n.s.). At 2 and 3 months of ages, none of the R6/1 mice displayed hind limb clasping, as expected in this mouse line (Naver et al. 2003; Lebreton et al. 2015).

### Spatial recognition in Y-maze

During the habituation phase, mice showed no preference for any of the two arms of the maze and no difference was observed among experimental groups at both ages (all effects, ns; Fig.5A). Genotype differences were instead detected on locomotor activity during this trial, and they depended on the age of testing [R6/1 genotype × age: *F*(1, 29) = 6.02, *P* < 0.05 and R6/1 × HDAC6 genotype × age: *F*(1, 29) = 7.38, *P* < 0.05; Fig.5B]. At 2 months of age, HDAC6 genotype resulted in an increase in locomotor activity in non-Tg mice, while an opposite (although nonsignificant) trend was observed in R6/1 animals [R6/1 × HDAC6 genotypes: *F*(1, 29) = 4.5, *P* < 0.05; post hoc: *P* < 0.05]. At 3 months of age, R6/1 mice showed lower levels of activity compared to non-tg littermates, an effect that was equally observed in HDAC6-WT and HDAC6-KO mice [R6/1 genotype effect: *F*(1, 29) = 7.39, *P* < 0.05].

**Figure 2:**
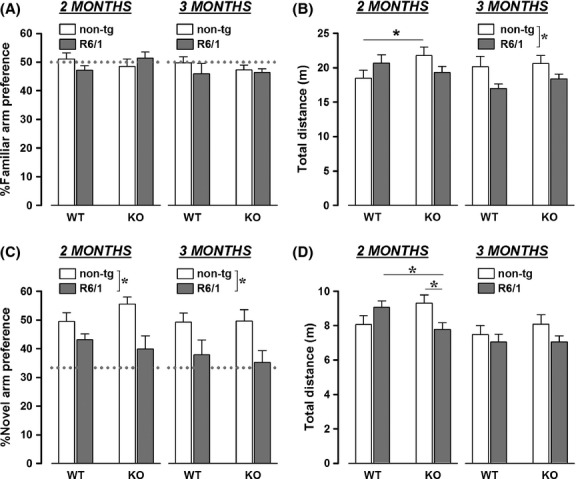
Behavioral effects of HDAC6 knockout in the Y-maze. Arm preference and traveled distance were evaluated both during the habituation phase (respectively, A and B) and on the subsequent test phase (C and D). Dotted gray line represents the chance level, *P < 0.05.

**Figure 3:**
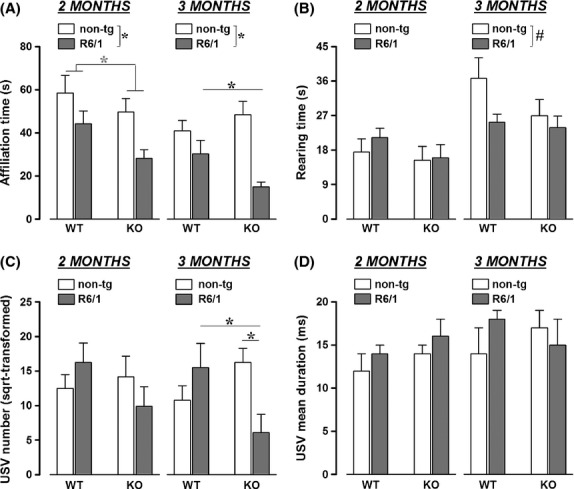
Effects of HDAC6 knockout on social behaviors in R6/1 mice. Affiliative behaviors (A), and nonsocial rearing (B) were assessed during a social interaction test as well as the number and duration of ultrasonic vocalizations (USVs). Affiliation time represents the time spent in active contact with the female partner. *P < 0.05, 
*P* = 0.05, ^#^*P* = 0.07.

**Figure 4:**
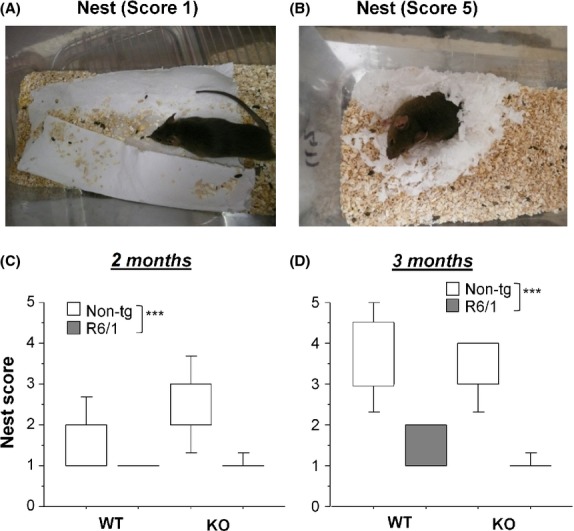
Effects of HDAC6 knockout on nest building in R6/1 mice. Two examples illustrate poor (score 1, A) and high (score 5, B) qualities of nest. Nest quality was scored at 2 months (C) and 3 months (D) of ages. ***P < 0.001 by Mann–Whitney test.

**Figure 5:**
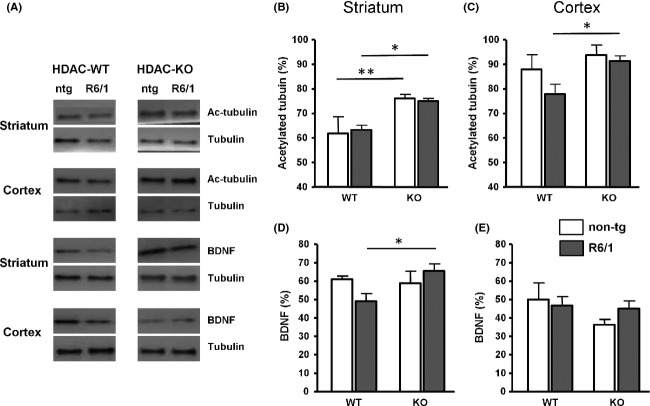
Effects of HDAC6 knock-out on tubulin acetylation and brain-derived neurotrophic factor (BDNF) protein levels. Representative western blots showing acetylated alpha-tubulin and mature BDNF in single (HDAC6-WT+R6/1, HDAC6-KO+HD-ntg), double mutant (HDAC6-KO+R6/1), and wt (HDAC6-WT+HD-ntg) mice for HDAC6 and HD mutations for both striatum and cortex (A). Acetylated tubulin was normalized to tubulin level and the ratios (in %) of acetylated tubulin/total amount of tubulin were shown for striatum (B) and cortex (C). The same normalization was performed for mature BDNF and relative BDNF levels were shown for striatum (D) and cortex (E) in single, double mutant, and wt mice. *P < 0.05, **P < 0.01 by Mann–Whitney test.

During the test phase, R6/1 mice, irrespective of their HDAC6 genotype, exhibited lower % of preference for novel arm, this at both ages [R6/1 genotype effect: *F*(1, 29) = 16.25, *P* < 0.001; Fig.5C]. Locomotor activity in this second trial closely resembled the pattern of results observed during the habituation phase: again, genotype differences emerged here but mainly depending on the testing age [HDAC6 genotype effect: *F*(1, 29) = 4.89, *P* < 0.05; age effect: *F*(1, 29) = 18.09, *P* < 0.001; R6/1 × HDAC6 genotypes × age: *F*(1, 29) = 3.14, *P* = 0.09; Fig.5D]. At 2 months of age, HDAC6 genotype induced opposite effects in non-tg and R6/1 mice, significantly decreasing locomotor activity in R6/1 animals as compared to non-tg mice [R6/1 × HDAC6 genotypes: *F*(1, 29) = 8.26, *P* < 0.01; post hoc: *P* < 0.05]. At 3 months of age, there was no difference among groups in locomotion [all effects: ns]: the effect of R6/1 genotype observed during the first trial disappeared on the second, mainly because of the low activity levels of HDAC6-WT+HD-ntg, probably due to habituation across trials.

### Social behavior

As illustrated in Fig.5A, social affiliative behaviors were modulated by both R6/1 and HDAC6 genotypes [main effects, respectively: *F*(1, 30) = 17.45 and 2.92, *P* < 0.001 and *P* = 0.09], and social behaviors changed with age in general [age effect: *F*(1, 30) = 16.01, *P* < 0.001]. Separate analyses indeed revealed that, at 2 months of age, both R6/1 and HDAC6 genotypes reduced the time spent in active contact with a partner [main effects, respectively: *F*(1, 30) = 8.47 and 4.06, *P* < 0.01 and *P* = 0.05], while at the age of 3 months HDAC6 genotype clearly exacerbated the effects of R6/1 mutation [main effect of R6/1 genotype: *F*(1, 30) = 20.03, *P* < 0.001 and interaction R6/1 × HDAC6 genotypes: *F*(1, 30) = 5.4, *P* < 0.05].

Differences were also observed on nonsocial activities (Fig.5B), i.e., rearing behavior: R6/1 genotype, as compared to their non-tg littermates irrespective of their HDAC6 genotype, reduced the time spent rearing and again this effect depended on the age of testing [R6/1 genotype x age effect: *F*(1, 30) = 4.08, *P* = 0.05]. R6/1 mice showed slightly lower levels of rearing activity at 3 months of age [main R6/1 genotype effect: *F*(1, 30) = 3.56, *P* = 0.07], while no group difference was detected at 2 months [all effects, ns].

The analysis of ultrasonic vocalizations (USVs) during the social interaction test led to even more marked effects of the HDAC6 genotype (Fig.5C and D). Indeed, while the R6/1 mutation alone did not alter ultrasonic communication, the genetic deletion of HDAC6 induced a reduction in the number of USVs emitted, an effect that was observed only in R6/1 mice [R6/1 × HDAC6 genotype interaction: *F*(1, 32) = 4.68, *P* < 0.05] and depended on the testing age [R6/1 × HDAC6 genotypes × age: *F*(1, 32) = 4.1, *P* = 0.05]. Double mutant mice emitted less USVs compared to all other groups only at 3 months of age [R6/1 × HDAC6 genotype interaction: *F*(1, 32) = 7.41, *P* < 0.05], while no significant group difference was observed at 2 months.

A qualitative analysis of USVs was also conducted, taking into account the mean duration of the vocalizations (Fig.5D). This analysis was obviously performed on the data of vocalizing mice, that is, excluding those animals that were not emitting any USV: these included 2 (of 8) HDAC6-WT+R6/1 and 6 (of 12) HDAC6-KO+R6/1 mice. No effect of either R6/1 or HDAC6 genotype was detected on USV duration, and no age difference was observed [all effects, ns].

### Nesting behavior

R6/1 mice, irrespective of their HDAC6 genotype, constructed nests of poorer quality as compared to non-tg littermates at both 2 months (Mann–Whitney test, *U* = 46, *U*′ = 270, *P* < 0.001, Fig.5C) and 3 months (Mann–Whitney test, *U* = 4, *U*′ = 316, *P* < 0.0001, Fig.5D). HDAC6-KO mice, irrespective of their HD genotype, were not different from their WT counter parts in their nesting abilities at neither age (*U* = 142, *U*′ = 178, n.s. at 2 months, *U* = 119, *U*′ = 201, n.s. at 3 months). Pairwise comparison revealed that both HDAC6-WT+R6/1 and HDAC6-KO+R6/1 mice constructed nests of poorer quality than HDAC6-WT+HD-ntg (*P* < 0.01 for both comparisons by Mann–Whitney test) and HDAC6-KO+HD-ntg mice (*P* < 0.001, for both comparisons by Mann–Whitney test) at 2 months and 3 months (*P* < 0.001 for four comparisons by Mann–Whitney test). In addition, HDAC6-WT+HD-ntg mice significantly improved the nest quality with age (*P* < 0.001, Mann–Whitney test), while such effect of maturation or “learning” was not present for the three remaining groups.

### Tubulin acetylation and BDNF

Genetic deletion of HDAC6 effectively increased ratio of acetylated alpha-tubulin versus total amount of tubulin of striatum in both R6/1 (Mann–Whitney test, *U* = 0, *U*′ = 15, *P* = 0.02) and ntg littermates (*U* = 0, *U*′ = 35, *P* = 0.005, Fig.5B), while such increase in cortex was found only in R6/1 mice (*U* = 2, *U*′ = 33, *P* = 0.012) (Fig.5C). Among HDAC6-WT mice, R6/1 mice, as compared to ntg counterpart, displayed a trend toward a diminution of striatal mature BDNF (*U* = 2, *U*′ = 13, *P* = 0.10, Fig.5D). This decrease in R6/1 mice was completely reversed by HDAC6 knockout such that BDNF level in double mutant mice was significantly elevated as compared to single-mutant HDAC6-WT+R6/1 mice (*U* = 4, *U*′ = 31, *P* = 0.03). Such variations of BDNF levels were not observed in cortex (Fig.5E).

## Discussion

HDAC6 inhibition has been shown to be beneficial for cell protection and survival by enhancing axonal transport of neurotrophic factors during aging and neurodegenerative diseases such as Alzheimer’s disease, Parkinson’s disease, and Huntington’s disease (Govindarajan et al. 2013; Simoes-Pires et al. 2013). Furthermore, impaired mitochondrial transport and the elimination of protein aggregates among other cellular processes are common abnormalities in several neurodegenerative diseases and are linked to both deacetylase and ubiquitin ligase activities of HDAC6 (Simoes-Pires et al. 2013). As such, experiments reducing HDAC6 produced symptomatic improvement in Charcot–Marie–Tooth disease (d’Ydewalle et al. 2011; Taes et al. 2013), amyotrophic lateral sclerosis (Taes et al. 2013), Parkinson disease (Outeiro et al. 2007; Du et al. 2010) as well as Alzheimer’s disease (Govindarajan et al. 2013). For example, the loss of HDAC6 alleviated the impairment of associative and spatial memory formation associated with the recovery of the deficits in mitochondrial trafficking induced by toxic *β*–amyloid (Kim et al. 2012; Govindarajan et al. 2013). Hence, we hypothesized that the genetic deletion of HDAC6 might rescue or at least retard early cognitive and psychiatric-like impairments in R6/1 mice.

In contrast, we observed that the deletion of HDAC6 did not ameliorate any of the major early HD phenotypes: it did not affect the body weight loss, the cognitive impairments, and nest-building deficits shown by R6/1 mice at both 2 and 3 months, while it actually worsened social abnormalities observed at 3 months of age. Interestingly, the combination of the HDAC6-KO and R6/1 genotype induced some HD-like phenotypes that were not present in single R6/1 mice, that is, hypolocomotion in the Y-maze at 2 months of age and a reduction in the number of USVs emitted at 3 months. These behavioral changes were accompanied by increased levels of tubulin acetylation in both striatum and cortex as well as BDNF protein level in striatum of R6/1 mice.

Our BDNF and behavioral data are at odds with previous investigations reporting that manipulations including genetic overexpression of BDNF, pharmacological treatments, or environmental enrichment, all enhancing BDNF levels in HD transgenic mice, produced notable alleviation of HD phenotypes (Spires et al. 2004; Zuccato et al. 2005; Peng et al. 2008; Xie et al. 2010). In addition, BDNF knockout mice have shown a similar phenotype to that observed in the mouse models of HD (Baquet et al. 2004; Strand et al. 2007). The reasons for the absence of improvement even with increased level of striatal BDNF in our transgenic mice could be multiple. One reason would be that HDAC6 owns different cellular targets and functions. For example, inhibition of HDAC6 acetylates and disrupts the chaperone function of heat shock protein 90 important for normal cell function (Bali et al. 2005). HDAC6 reduction also contributes to the accumulation of ubiquitinated proteins that need to be degraded (Iwata et al. 2005). It is thus trivial to say that the effects of HDAC6 reduction other than BDNF changes have greatly contributed to complex phenotypes observed in our study (also see below).

The behavioral phenotypes observed here in single HD mutant (i.e., HDAC6-WT+R6/1) mice confirm previous studies on early nonmotor deficits in this mouse line (Naver et al. 2003; Pang et al. 2006; Nithianantharajah et al. 2008; Pietropaolo et al. 2011; Lebreton et al. 2015), although with some subtle differences in the age of their appearance, may be due to different genetic backgrounds (i.e., B6/sv129 as compared to B6/CBA or C57bl/6 in the previous studies) used. More precisely, spatial recognition deficits in the Y-maze appeared at 8 weeks in our study, that is, slightly earlier than what previously described (Nithianantharajah et al. 2008), while the social deficits were not as marked as previously observed in 3-month-old R6/1 mice (Pietropaolo et al. 2011). Yet, our results confirm the presence of early nonmotor behavioral deficits that resemble the psychiatric HD symptoms of reduced behavioral flexibility/executive functions and social interaction deficits. It could be noted that the appearance of these behavioral deficits in R6/1 mice seems independent from that of motor impairments: abnormalities in spatial recognition, nest building, and social interaction are present in R6/1 mice already at 2 months of age, when no hypolocomotion is detected when assessed in the Y-maze. Our data also show a novel early phenotype of R6/1 mice, that is, deficit in nest-building behavior. Interestingly, this fine sensorimotor and species-specific behavior is also altered in genetic mouse models of other neurodegenerative disorders, for example, Parksinsons (Paumier et al. 2013), Alzheimer diseases (Filali and Lalonde 2009) and hippocampal-lesioned mice (Deacon and Rawlins 2005).

The lack of effect of HDAC6 deletion on weight loss, spatial recognition deficits, and nest building are in agreement with previous observation on the motor phenotype of R6/2 mice by Bobrowska et al. (2011), who suggested to discard HDAC6 as a therapeutic target for HD. Our results suggest the potentially aggravating role of HDAC6 in HD pathology: HDAC6 deletion not only exacerbated the social impairments shown by R6/1 mice but it also induced ultrasonic deficits. Both effects were observed at 3 months of age, although an effect of the double mutant genotype was detected on locomotion in the Y-maze already at 2 months.

Factors that aggravated specifically the social behavioral deficits might be related to the neuroanatomical expression of HDAC6 in the mouse brain and maybe throughout body. HDAC6 is shown to be highly expressed in the amygdala, hippocampus, and locus coeruleus in addition to the striatum and cortex, regions severely affected by HD mutation (Khochbin et al. 2001; de Ruijter et al. 2003; Mai et al. 2005; Simoes-Pires et al. 2013), and important for social behaviors (Weiskrantz 1956; Aggleton and Passingham 1981; Adolphs 1999, 2010; Goodson and Thompson 2010; Insel 2010; Kemp et al. 2013). It is thus probable that HDAC6 deletion in these regions may induce social withdrawal in R6/1 mice.

Our results provide further evidence that targeting HDAC6 in HD may not be a favorable therapeutic strategy. This is in discrepancy to its reported role in other aggreopathies such as AD, and may be attributed to the fact that the HDAC6 function is critical for autophagy that plays an important role in HD pathogenesis (Iwata et al. 2005; Pandey et al. 2007a,b). It is thus likely that the negative cellular processes induced by the HDAC6 knockout in particular regions such as the prefrontal cortex, striatum, or amygdala could be directly associated with exacerbated social defects in our double mutant mice. The absence of HDAC6 manipulation effects on other behavioral domains in R6/1 mice could also be explained by compensatory processes taking place in double mutant mice.

In conclusion, the present results may go against the potential therapeutic impact of HDAC6 modulation for HD, but demonstrate a specific role of HDAC6 in the emergence of social HD-like deficits that requires further investigation. Future studies involving time or tissue-specific knockout of the HDAC6 or intracerebral injections of a new generation of HDAC6 inhibitors (Jochems et al. 2014; Lee et al. 2013) will be necessary to clarify the specific molecular mechanisms underlying the role of this class of HDAC for HD pathophysiology.
